# Clinical and radiographic differentiation of lung nodules caused by mycobacteria and lung cancer: a case–control study

**DOI:** 10.1186/s12879-015-1185-4

**Published:** 2015-10-28

**Authors:** Cesar J. Figueroa, Elyn Riedel, Michael S. Glickman

**Affiliations:** Department of Medicine, Infectious Diseases, Memorial Sloan Kettering Cancer Center, 1275 York Avenue, New York, NY 10065 USA; Department of Epidemiology and Biostatistics, Memorial Sloan Kettering Cancer Center, 1275 York Avenue, New York, NY 10065 USA; Immunology Program, Memorial Sloan Kettering Cancer Center, 1275 York Avenue, New York, NY 10065 USA

**Keywords:** Pulmonary Mycobacteria, Mycobacterial Lung Nodules, Pulmonary Nodule, Positron-Emission Tomography, Lung Neoplasms

## Abstract

**Background:**

Lung nodules caused by mycobacteria can resemble lung cancer on chest imaging. The advent of lung cancer screening with low-dose Computed Tomography is accompanied by high false-positive rates, making it necessary to establish criteria to differentiate malignant from benign nodules.

**Methods:**

We conducted a retrospective case–control study of 52 patients with mycobacterial lung nodules and 139 patients with lung cancer, diagnosed between 2010 and 2012. We compared clinical and radiographic characteristics to identify predictors of disease by univariate and multivariate analysis. The discriminatory power of maximum Standardized Uptake Values from Positron-Emission-Tomography was also evaluated.

**Results:**

Several variables were correlated with a diagnosis of mycobacterial infection or lung cancer on univariate analysis. Such variable include smoking status and history, lesion size and imaging evidence of tree-in-bud opacities, lymphadenopathy or emphysema on computed tomography. Upon author consensus, the most clinically-relevant variables were selected to undergo multivariate analysis. A history of current or former smoking [OR 4.4 (95 % CI 1.2–15.6) and 2.7 (95 % CI 1.1–6.8), respectively *P* = 0.04] was correlated with diagnoses of lung cancer. Contrarily, the presence of tree-in-bud opacities was less likely to be correlated with a diagnosis of malignancy [OR 0.04 (95 % CI 0.0–1.0), *P* = 0.05]. Additionally, higher maximum standardized uptake values from positron emission tomography were associated with malignancy on multivariate analysis [OR 1.1 (95 % CI 1.0–1.2), *P* = 0.04]; but the accuracy of the values in differentiating between diseases was only 0.67 as measured by the area under the curve. Lesion size was not independently associated with diagnosis [OR 0.5 (95 % CI 0.2–1.2), (*P* = 0.12)].

**Conclusions:**

Establishing the likelihood of malignancy for lung nodules based on isolated clinical or radiographic criteria is difficult. Using the variables found in this study may allow clinicians to stratify patients into groups of high and low risk for malignancy, and therefore establish efficient diagnostic strategies.

**Electronic supplementary material:**

The online version of this article (doi:10.1186/s12879-015-1185-4) contains supplementary material, which is available to authorized users.

## Background

Recent clinical trials have demonstrated substantial mortality benefit from using low-dose chest Computed Tomography to screen for lung cancer in high-risk patients [1, 2]. Most U.S. guidelines now recommend this strategy [3–5]. Despite these advantages; screening with computed tomography frequently identifies non-malignant lung lesions, resulting in false positive rates that are as high as 70 % [2, 6–8]. In the large, randomized National Lung Screening Trial, many lung nodules were ultimately nonmalignant, but the eventual histologic diagnoses of these nodules have not been reported. Since the implementation of routine screening of lung cancer will most likely be accompanied by an increase in the rates of detection of benign nodules, including those cause by mycobacteria, a condition frequently encountered by infectious diseases specialists, differentiating malignant from infectious nodules before invasive lung sampling, will become increasingly important in the years to come [1].

The clinical spectrum of mycobacterial lung disease is broad and ranges from a state of asymptomatic-carrier based on culture positivity, to a wasting illness with cavitary lung lesions that mimic tuberculosis. The radiographic spectrum of mycobacterial lung infection on chest imaging is broad and includes solitary pulmonary nodules [9, 10] that mimic malignancy [11, 12], in addition to the more classic nodular-bronchiectatic and cavitary presentations. Although descriptions of the manifestations of mycobacterial lung disease on Computed Tomography (CT) [9, 13], and Positron-Emission Tomography (PET) [14, 15] exist; a direct comparison between radiographic and clinical characteristics of lung nodules caused by mycobacteria and cancer has not been performed. In this study we compared such characteristics in consecutive patients evaluated for suspicious lung lesions in our center.

Our goal was to describe clinical and radiographic differences between patients with mycobacterial or malignant lung nodules that can serve as predictors of the etiology of suspicious nodules before tissue sampling is performed. We identify relevant clinical and radiographic variables and determine their association with the occurrence of each diagnosis. We believe such variables can assist clinicians evaluating the etiology of pulmonary nodules detected incidentally or during screening for lung cancer.

## Methods

Memorial Sloan Kettering Cancer Center is a 432-bed, tertiary-care center in New York City that serves patients suffering from lung malignancies. We conducted a case–control; retrospective study that included patients diagnosed with suspicious pulmonary lesions between April 2010 and April 2012, whose diagnosis was not a result of lung cancer screening. The Memorial Sloan Kettering Cancer Center Institutional Review Board approved this study and a waiver for the need to obtain informed consent from patients was issued (approval#WAC-0150-12 for review of existing data). Patient records and information was anonymized and de-identified prior to analysis. The study was conducted in accordance with the amended declaration of Helsinki. Subjects were identified according to the following definitions:

### Nodular lung disease

Defined as the presence of one or more lesions within and surrounded by lung parenchyma [16] on chest imaging. Patients with lesions ten or more millimeters in longest diameter were included. Lesions 30 mm or longer in biggest diameter were further classified as lung masses.

### Mycobacterial nodular lung disease (cases)

Diagnostic criteria from the American Thoracic Society and Infectious Diseases Society of America for Non-Tuberculous Mycobacterial lung disease were used [17]. Patients with lesions caused by *Mycobacterium tuberculosis* were included and similar criteria were used. The case definition included 1) lung histopathology consistent with granulomatous inflammation; and 2) microbiologic evidence of mycobacterial infection with one or more of the following: a) Positive mycobacterial stain in lung tissue (Ziehl-Nielsen or Fite); b) Positive mycobacterial culture from lung tissue; c) positive mycobacterial culture from one bronchoalveolar lavage/wash or at least two sputum samples. Patients were excluded if malignancy was diagnosed on the same lung sample.

### Malignant nodular lung disease (controls)

Patients in this category included those who were diagnosed with primary lung cancer; excluding patients diagnosed with recurrent lung cancer (lung tumors diagnosed within the prior five years), or pre-existing lung cancer (lung tumors actively being treated at the time of diagnosis).

### Data collection

Patients were identified using electronic searches in various databases. A microbiology database search identified all patients with reports of positive mycobacterial cultures. Only patients with positive samples from lung tissue, bronchoalveolar lavage/wash or sputum were considered for inclusion. Searches within infection control and pathology databases identified patients with granulomatous inflammation and positive mycobacterial stains on histopathology, but whose cultures were negative or not performed. A search within the institutional tumor database identified patients diagnosed with lung cancer during the period of interest. Collected data on individual patients included age, gender, race, height, weight, body mass index; dates of chest imaging, tumor diagnosis; diagnostic procedure(s) and their respective dates.

A review of electronic medical records identified additional variables of interest. Patients were considered symptomatic if they had any type of respiratory or relevant non-respiratory symptoms (fever, weight loss or night sweats) at the moment of diagnosis or during six months prior. Patients were categorized as born in the United States or elsewhere. Imaging variables were collected from reports of CT and PET performed closest to the date of diagnosis. Variables from tomography included number and size of lung lesions, presence of cavitation, spiculation or surrounding ground glass opacities. The presence of abnormalities in the surrounding lung parenchyma and thoracic structures was recorded, including: bronchiectasis, tree-in-bud inflammation, lymphadenopathy, atelectasis, emphysematous lung, and pleural effusion. For PET, we recorded whether lesions were hypermetabolic and if so, the maximum standardized uptake value (SUV Max) of the dominating lesion.

### Microbiology

Processing of specimens included an initial Auramine-Rhodamine smear; followed by inoculation into plates with Middlebrook/7H11 agar, and a mycobacterial growth indicator tube. Plates were incubated at 35 °C with 5-7 % CO2; and mycobacterial growth indicator tubes were placed on a BACTEC™ MGIT™ instrument for six weeks. Any resulting growth was smeared and stained with the Kinyoun technique; followed by probe testing (*M. tuberculosis*-complex, *M. avium*-complex, *M. kansasii* and *M. gordonae*; AccuProbe, Gen-Probe™). Isolates that were negative by all four probes were sent for 16S rRNA sequencing for final identification.

### Statistical analysis

Univariate associations between case/control groups and clinical and radiographic characteristics were evaluated using Fisher’s exact test for categorical variables, Mantel-Haenszel test for trend for ordinal variables and the Wilcoxon rank sum test for continuous variables. Selected clinical and radiographic characteristics that were significantly associated with case/control group univariately were further examined in a multivariate logistic regression model. A receiver operating characteristic curve was generated to examine the accuracy of SUV max in differentiating between lung malignancy and mycobacterial infection. All calculations were performing using SAS version 9.2 (SAS Institute, Cary, NC).

## Results

During the period of interest, 534 positive mycobacterial cultures corresponding to 333 patients were reported by the microbiology laboratory. Infection control and pathology databases identified ten additional patients. An initial chart review excluded 291 patients. One hundred and sixty two lacked histopathological analysis and 90 had alternative diagnoses on histopathological analysis including malignancy, non-mycobacterial infections (*Pneumocystis*, *Coccidioides*), pneumonitis, or inconclusive biopsy results.

During the same time period, 742 patients were diagnosed with lung cancer. Due to the large number of patients in the control group, a representative-random sample of 150 patients was generated by the statistician (ER). A subsequent review of electronic medical records excluded 11 patients. Four of them had pre-existing lung cancer and seven had recurrent lung cancer. One hundred and thirty-nine patients were included in the final analysis.

Baseline demographic characteristics of patients are summarized in Table 1. Patients from both groups were similar in terms of age, gender, place of birth and ethnicity. The majority of patients were female, and the most common ethnicity was non-Hispanic white. The median Charlson Comorbidity Index (CCI) was similar in both groups. However, Chronic Obstructive Pulmonary Disease (COPD) was more prevalent amongst patients diagnosed with lung cancer (23.0 % vs 7.7 %, *P* = 0.02). The majority of patients in both groups were asymptomatic at the time of diagnosis [thirty-one (59.6 %) in the cases group vs seventy-three (52.5 %) in the controls group, *P* = 0.42, Table 1]. For symptomatic patients, the most commonly reported symptoms are described in Table 1. Notably, among symptomatic patients there was no difference between cases and controls in the incidence of cough (*P* = 0.52).

**Table 1:** Baseline clinical and demographic characteristics

Variable	Mycobacterial lung disease (52)	Lung Cancer (139)
Age, y, median (range)	67 (4–91)	67 (38–90)
Male	17 (32.7)	65 (46.8)
Female	35 (67.3)	74 (53.2)
CCI, Median (IQR)	1 (0–2)	1 (0–2)
Race		
Non-Hispanic white	42 (80.8)	120 (86.4)
Asian	4 (7.7)	7 (5.0)
Hispanic	4 (7.7)	7 (5.0)
Black	1 (1.9)	5 (3.6)
Unknown	1 (1.9)	0
Place of birth		
United States	40 (77.0)	97 (70.0)
Non-United States	12 (23.0)	42 (30.0)
Symptom status		
Asymptomatic	31 (59.6)	73 (52.5)
Symptomatic	21 (40.4)	66 (47.5)
• Cough	24 (46.2)	72 (51.8)
• Dyspnea	10 (19.2)	49 (35.3)
• Hemoptysis	2 (3.8)	13 (9.4)
• Wheezing	2 (3.8)	8 (5.8)
• Self-reported fever	3 (5.8)	2 (1.4)
• Involuntary weight loss	6 (11.5)	11 (7.9)
• Chest pain	3 (5.8)	0
• Night sweats	2 (3.8)	1 (0.7)
Indication for imaging		
Symptom evaluation	21 (40.4)	66 (47.5)
Cancer surveillance	13 (25.0)	23 (16.5)
Incidental finding	18 (34.6)	50 (36.0)
Diagnostic procedure		
Thoracotomy and biopsy	29 (55.0)	51 (37.0)
Transthoracic lung biopsy	18 (35.0)	53 (38.0)
Bronchoscopy and transbronchial lung biopsy	5 (10.0)	35 (25.0)
Selected Comorbidities		
Hypertension	24 (46.2)	65 (46.8)
COPD	4 (7.7)	32 (23.0)
Diabetes	4 (7.7)	21 (15.1)
Connective tissue disorder	2 (3.8)	5 (3.6)
Asthma	1 (1.9)	9 (6.5)
Mitral valve prolapse	3 (5.8)	2 (1.4)

Data are presented as No. (%) unless otherwise specified. *Abbreviations*: *CCI* Charlson Comorbidity Index, *COPD* Chronic obstructive pulmonary disease

Procedures performed for lung tissue sampling are described in Table 1. The majority of patients in the mycobacterial group (28 patients, 55 %) underwent thoracotomy and lung wedge biopsy. This was followed by transthoracic lung biopsy (18 patients, 35 %) and transbronchial biopsy (5 patients, 10 %). One patient underwent a lobectomy. Patients in the lung cancer group underwent surgical and transthoracic lung biopsy at similar rates (51 patients, 37 % and 53 patients, 38 % respectively), and a minority underwent bronchoscopy and transbronchial lung biopsy (35 patients, 25 %).

Lung tissue sampling and histopathology analysis was performed in all of the patients diagnosed with mycobacterial disease. Core biopsies were available in forty-three patients, and cytological analysis of lung aspirate was available in the remaining nine patients. All patients with mycobacterial infection had evidence of granulomatous inflammation. Of those who had core biopsies, thirty-six had evidence of necrotizing granulomas (83.7 %), six had evidence of non-necrotizing granulomas (14 %) and one patient had evidence of both necrotizing and non-necrotizing granulomas (2.3 %). All the patients who had cytological analysis performed had cytological elements suggestive of granulomatous inflammation. Fite or Ziehl-Neelsen acid fast stains were performed in fifty one out of the fifty two available surgical specimens, and were positive in twenty three (45 %) patients, with Fite being the most common positive stain.

Of the types of Mycobacteria isolated from respiratory samples, *Mycobacterium avium-*complex (MAC) was the most common isolated species (81 %) followed by *Mycobacterium tuberculosis* (9 %)*, Mycobacterium xenopi* (4 %) and *Mycobacterium haemophilum* (4 %). In two cases (2 %), the Auramine-Rhodamine stain was positive but cultures were sterile. The majority of patients (79 %) had mycobacteria recovered from lung tissue, followed by bronchoalveolar lavage/wash (11 %) and induced sputum (8 %). Regarding the distribution of the types of lung cancer, adenocarcinoma was most common (68 %), followed by squamous cell carcinoma (14 %), small cell carcinoma (7 %), large cell carcinoma (3 %), carcinoid tumor (3 %), others (including adenosquamous, pleomorphic and pseudosarcomatous carcinoma) (3 %) and non-small cell carcinoma (2 %).

Table 2 summarizes the radiographic findings in both groups. Most of the patients in both groups had solitary nodules within a single lobe [40 (76.9 %) patients in the mycobacterial group and 121 (87.1 %) patients in the lung cancer group]. Although there was a slightly higher percentage of patients with solitary nodules in the lung cancer group, this difference was not statistically significant (*P* = 0.12, Table 2). However; mycobacterial lung lesions corresponded more commonly to lung nodules (<3 cm); whereas lesions caused by lung cancer corresponded more commonly to masses (≥3 cm) (*P* < 0.0001, Table 2). The median maximum diameter of mycobacterial lesions was also shorter than malignant lesions (20 mm vs 30 mm respectively, *P* = 0.0005, Table 2).

**Table 2:** Radiographic findings on chest Computed Tomography and Positron-Emission-Tomography

Finding	Category	Mycobacterial lung disease (52)	Lung cancer (139)	*P*-value
Multiple lobes affected	No	40 (76.9)	121 (87.1)	0.12
	Yes	12 (23.1)	18 (12.9)	
Number of lobes affected^a^	Two	6 (50.0)	14 (77.8)	0.001
	Three	0	4 (22.2)	
	Four	5 (41.7)	0	
	Five	1 (8.3)	0	
Localization of dominating lesion^b^	Left lower lobe	6 (15.0)	11 (9.1)	
	Left upper lobe	8 (20.0)	30 (24.8)	
	Right lower lobe	3 (7.5)	25 (20.7)	
	Right middle lobe	5 (12.5)	8 (6.6)	
	Right upper lobe	18 (45.0)	47 (38.8)	
Size of Dominating lesion	Maximum length in mm (Median, IQR)	20 (14–26)	30 (19–49)	0.0005
Lesion type	Nodule	43 (82.7)	67 (48.2)	<0.0001
	Mass	9 (17.3)	72 (51.8)	
Positron-Emission-Tomography findings				
Examined variable	Category	Mycobacterial Lung Disease (52)	Lung Cancer (139)	*P*-value
PET available	Yes	43 (82.7)	137 (98.6)	
	No	9 (17.3)	2 (1.4)	
Hypermetabolic lesion	Yes	42 (97.7)	125 (92.6)	
	No	1 (2.3)	10 (7.4)	
	Result not available	0	2	
SUV Max	Median (IQR)	6.5 (4.0–8.7)	9.5 (4.9–14.5)	0.001
Lung parenchyma and thoracic structures				
	Total findings			
Bronchiectasis	22 (11.5)	13 (25.0)	9 (6.5)	0.001
Intrathoracic lymphadenopathy	64 (33.5)	8 (15.4)	56 (40.3)	0.001
Cavitary lesion	19 (9.9)	7 (13.5)	12 (8.6)	0.41
Atelectasis	31 (16.2)	4 (7.7)	27 (19.4)	0.08
Emphysematous lung	59 (30.9)	9 (17.3)	50 (36.0)	0.01
Perilesional ground glass	21 (11)	3 (5.8)	18 (12.9)	0.20
Spiculated lesion	44 (23)	15 (28.8)	29 (20.9)	0.25
Tree-in-bud opacities	9 (4.7)	9 (17.3)	0	<0.0001
Pleural effusion	15 (7.9)	1 (1.9)	14 (10.1)	0.07

Data are presented as No. (%) unless otherwise specified. *Abbreviations*: *SUV Max* Maximum Standardized Uptake Value, *PET* Positron-Emission Tomography

^a^In patients with multiple lobes affected

^b^In patients with a single lobe affected

Lesions caused by mycobacteria were commonly associated with bronchiectasis (25.0 % vs 6.5 %, *P* = 0.001, Table 2) and a tree-in-bud pattern in the surrounding lung parenchyma (4.7 % vs 0, *P* < 0.0001, Table 2). In contrast, intrathoracic lymphadenopathy (40.3 % vs 15.4 %, *P* = 0.001, Table 2) and emphysema (36.0 % vs 17.3 %, *P* = 0.01, Table 2) were more commonly seen in patients diagnosed with lung cancer. Atelectasis and pleural effusions were more commonly found in lung cancer patients; however this difference did not reach statistical significance.

Maximum standardized uptake values from PET were available for 43 (82.7 %) cases and 137 (98.5 %) controls. Lesions caused by lung cancer had a higher median SUV max value than those caused by mycobacteria (SUV max = 9.5 vs SUV max = 6.5, respectively; *P* = 0.001, Table 2). Results of a receiver operating characteristic curve of SUV max values from PET scan are shown in Additional file 1. The area under the curve was 0.67, reflecting a poor level of accuracy for differentiating lesions caused by mycobacteria and lung cancer. However, none of the lesions caused by Mycobacteria had SUV max values ≥ 16.

**Fig. 1:**
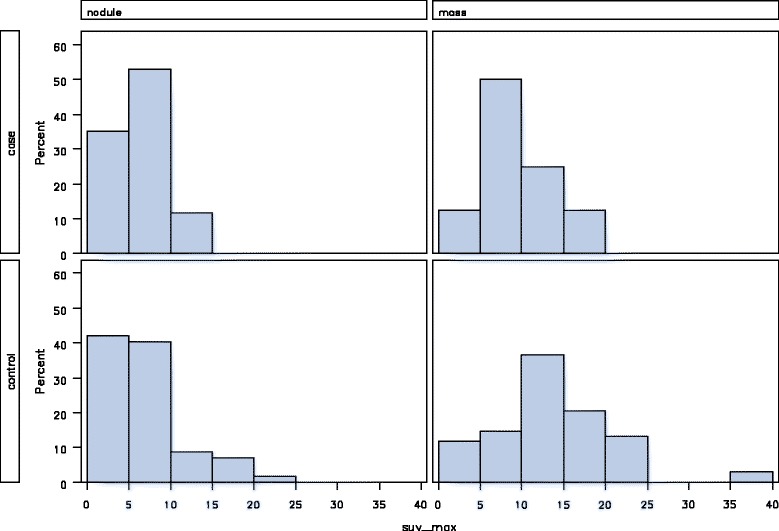
Maximum standardized uptake value by disease and lesion type

**Fig. 2:**
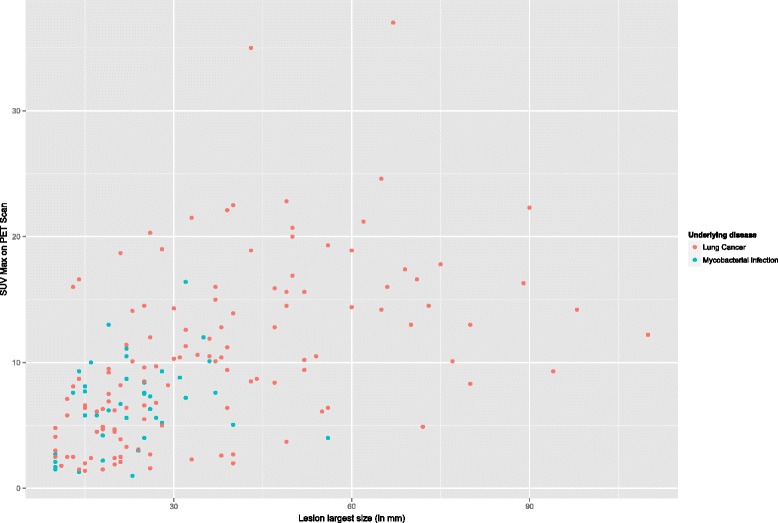
Scatterplot of maximum standardized uptake values and largest lesion size (in millimeters)

Analysis of the relationship between SUV max value and lesion size showed no differences between the median SUV max of nodular lesions caused by lung cancer or mycobacterial infection [median SUV max 6 (IQR 3–8.1) vs 6.2 (IQR 3–8.5), respectively]. However, the median SUV max of masses caused by lung cancer was higher than masses caused by mycobacteria [median SUV max 12.9 (IQR 9.5–16.8) vs 8.2 (IQR 6.1–11.1) respectively]. A graphic representation of the distribution of SUV max values stratified by lesion (nodule vs mass) and underlying disease types (mycobacterial infection vs cancer) is shown in Fig. 1. A scatterplot of SUV max and corresponding lesion size demonstrated no relationship between the degree of hypermetabolism and the size of any given lesion, Fig. 2. These results therefore suggest that the observed higher degree of hypermetabolism exhibited by malignant nodules cannot be explained solely by the observed difference in size between neoplastic and mycobacterial lesions.

Analysis of clinical variables is shown in Table 3. Patients with lung cancer were more likely to be current smokers compared to patients diagnosed with mycobacterial lung disease (7.7 % of cases vs 27.3 % of controls, *P* = 0.005, Table 3). The cumulative tobacco exposure was also significantly higher in patients with lung cancer compared to those with mycobacterial infection (median 40 pack-years vs 23 pack-years, respectively, *P* < 0.0001, Table 3). Patients with lung cancer had a higher average body mass index compared to subjects with mycobacterial lung nodules (Mean BMI 27.3 kg/m^2^ vs 25.1 kg/m^2^, *P* = 0.01, Table 3). Case-patients were more likely to have a history of cancer than controls (50.0 % vs 25.9 %, *P* = 0.003, Table 3); however, the majority of patients with any history of cancer in both groups had no evidence of active malignancy at the time of diagnosis (76.9 % in the mycobacterial lung disease group vs 77.8 % in the lung cancer group, *P* = 1.0, Table 3).

**Table 3:** Univariate and multivariate analysis of selected clinical and radiographic variables

	Univariate analysis				Multivariate analysis	
Risk Factor	Category	Mycobacterial lung disease (52)	Lung Cancer (139)	*P*-value	OR for lung cancer vs mycobacterial lung disease (95 % CI)^d^	*P*-value
Smoking status	Current	4 (7.7)	38 (27.3)	0.005	4.4 (1.2–15.6)	0.04
	Former	33 (63.5)	78 (56.1)		2.7 (1.1–6.8)	
	Never	15 (28.8)	23 (16.5)		1	
Cumulative smoking^a^	Median (IQR)	23 (5–39)	40 (30–58)	<0.0001		
Body mass index	Mean (STD)	25.1 (4.9)	27.3 (5.3)	0.01		
History of cancer	No	26 (50.0)	103 (74.1)	0.003		
	Yes	26 (50.0)	36 (25.9)			
Number of tumors^b^	0	26 (50.0)	103 (74.1)	0.004		
	1	23 (44.2)	29 (20.9)			
	2+	3 (5.8)	7 (5.0)			
Active cancer^c^	No	20 (76.9)	28 (77.8)	1.0		
	Yes	6 (23.1)	8 (22.2)			
Lesion type	Nodule (<3 cm)	43 (82.7)	67 (48.2)	<0.0001	0.5 (0.2–1.2)	0.12
	Mass (≥3 cm)	9 (17.3)	72 (51.8)		1	
SUV max	Median (IQR)	6.5 (4.0–8.7)	9.5 (4.9–14.5)	0.001		
	Per unit increase				1.1 (1.0–1.2)	0.04
Tree-in-bud opacities in CT	No	43(82.7)	139 (100)	<0.0001	1	0.05
	Yes	9 (17.3)	0		0.04 (0.0–1.0)	

Data are presented as No. (%) unless otherwise specified. *Abbreviations*: *BMI* Body Mass Index, *OR* Odds Ratio, *CI* Confidence Interval, *SUV Max* Maximum Standardized Uptake Value

^a^Expressed in pack-years

^b^In patients with a history of cancer

^c^Of those patients with a history of cancer

^d^Results presented as adjusted OR

Upon authors’ consensus, variables thought to be most clinically-relevant including smoking status at the time of diagnosis, lesion type (nodule vs mass), SUV max value, and tree-in-bud in the surrounding parenchyma, were selected for multivariate analysis. The number of patients in the study did not allow inclusion of all the significant variables from univariate analysis for multivariate analysis. Results are shown in Table 3 and are presented as adjusted odds ratios (OR). Factors associated with increased odds of a lesion being lung cancer were history of smoking at the time of diagnosis (current smoker vs never smoker: OR 4.4, 95 % CI 1.2–15.6; former smoker vs never smoker: OR 2.7, 95 % CI 1.1–6.8; *P* = 0.04, Table 3); and unit increases of SUV max value of dominating lesions (per unit increase in SUV max: OR 1.1, 95 % CI 1.0–1.2, *P* = 0.04, Table 3). In contrast, the odds of receiving a diagnosis of lung cancer were significantly lower in the presence of a tree-in-bud inflammatory pattern in the surrounding parenchyma (OR 0.04, 95 % CI 0.0–1.0, *P* = 0.05). Lesion size was not independently associated with lung cancer (*P* = 0.12).

## Discussion

Diagnosing mycobacterial lung nodules prior to tissue sampling is challenging due to several factors, including the relative lack of awareness of this type of mycobacterial infection. For the fibrocavitary and nodular/bronchiectatic forms of the disease, clinical, radiographic and microbiologic criteria can be more easily applied [17]. In the case of nodular disease, the frequent absence of systemic or pulmonary symptoms impede collection of microbiologic data, which would provide early clues to the possibility of mycobacterial infection as the etiology of lung nodules [18]. Such microbiologic data is often not collected in cases of purely nodular lung disease due to the broad differential diagnosis and the priority of excluding lung cancer. Similar to previous studies, all mycobacterial lung lesions in our series were suspicious for malignancy based on their radiographic appearance [12, 19, 20]. There have been no previously described clinical and radiographic criteria that can reliably differentiate malignant lung lesions from those caused by mycobacteria [20]. Identifying such factors will become increasingly important as computed tomography screening for lung cancer becomes more widespread and more false-positive results are generated [21].

Mycobacteria are common causative organisms of lung nodules as reflected by previous reports that describe infection as a common etiology (~20 %) of suspicious lung nodules, with around 25 % of such infections corresponding to mycobacteria [22]. Additionally, an increase in the incidence and prevalence of mycobacterial infections in different areas of the world has been described [23–25]; likely due to increased awareness of their role as human pathogens [24]; and possibly due to environmental and pathogen-specific factors [26]. Nonetheless, lung cancer screening undoubtedly will be associated with further increase in the numbers of mycobacterial infections, making it necessary to better understand the manifestations of this type of disease. In our study, we describe clinical and radiographic parameters that may help clinicians suspect mycobacterial infection or malignancy prior to lung tissue sampling.

Patients with mycobacterial lung disease were commonly former or never smokers and had a comparative lower tobacco exposure, compared to patients with lung cancer. The latter also had a higher prevalence of COPD. This scenario is in accordance with clinical guidelines that recommend lung cancer screening in patients with a tobacco exposure ≥ 30 pack-years [5, 7]. Additionally, most cases of mycobacterial infection were diagnosed during cancer surveillance or incidentally, in patients who were asymptomatic.

Several radiographic characteristics did differentiate between mycobacterial and malignant nodules. Malignancy commonly corresponded to larger lesions, but this correlation was only observed in a univariate analysis. Changes in the lung parenchyma and thoracic structures provided more significant clues to differentiate mycobacterial from neoplastic lesions. Although, a tree-in-bud inflammatory pattern on the lung parenchyma was only present in a minority of patients with mycobacterial disease, it was never found in association with a malignant nodule. This association was significant in both univariate and multivariate analyses and should be considered a clue for suspecting mycobacterial infection.

Even though PET scan has been postulated as a potential tool for the diagnosis and monitoring of infectious and malignant lesions [27–29], in our study, it lacked specificity for differentiating neoplasia from mycobacterial infection. Such findings differ from previous studies that describe PET as a useful tool in differentiating benign from malignant lesions [14, 30]. We encountered that SUV max values ≥ 16 had the highest specificity and positive predictive value for diagnosing lung cancer; and it is possible that studies that include other types of infection may show different results regarding PET performance. This underlies the importance of improving the specificity of chest imaging during lung cancer screening, in order to efficiently differentiate lung malignancies from benign lesions.

Strengths of our study include the availability of histopathology, culture and imaging results for all patients, allowing inclusion of proven cases of mycobacterial infection. The nature of our institution as a reference center for the diagnosis of lung cancer also favored a higher number of cases of mycobacterial lung nodules. Limitations included the retrospective nature of the study, which prevented a more extensive assessment of patients’ symptoms; as well as revision of existing imaging results.

## Conclusions

In our study, we found that individual clinical and imaging characteristics are poor predictors of cancer or mycobacterial disease in patients with lung nodules. Our data suggests that a diagnosis of lung cancer would be favored for large lesions; occurring in current smokers; and that have very high SUV max values on PET. In contrast, mycobacterial infection should be suspected when a tree-in-bud pattern is seen in association with lung nodules in patients with a less significant degree of smoking. Larger clinical studies are needed in order to establish additional clinical and radiographic parameters that help establish the likelihood of malignancy of newly diagnosed lung nodules, and subsequently stratify the risk of individual patients, allowing a rapid and efficient diagnosis. As lung cancer screening becomes more prevalent, these criteria can be refined further as more pathologic data become available on asymptomatic lung nodules detected by tomography.

## Additional file

Additional file 1:
**Receiver operating characteristic curve for maximum standardized uptake values on differentiating between mycobacterial lung disease and lung cancer.** (PDF 67 kb)

## Abbreviations

OR: Odds ratio
CI: Confidence interval
CT: Computed tomography
PET: Positron-Emission tomography
SUV Max: Maximum standardized uptake value
CCI: Charlson comorbidity index
COPD: Chronic obstructive pulmonary disease
MAC: *Mycobacterium avium* complex
IQR: Interquartile range
