# Supplementary material for: Clinical and radiographic differentiation of lung nodules caused by mycobacteria and lung cancer: a case–control study
Source: BMC Infect Dis. 2015 Oct 28;15:482. doi: 10.1186/s12879-015-1185-4 (PMC4625432; doi:10.1186/s12879-015-1185-4)
Supplement: Additional file 1: — Receiver operating characteristic curve for maximum standardized uptake values on differentiating between mycobacterial lung disease and lung cancer. (PDF 67 kb) [file 12879_2015_1185_MOESM1_ESM.pdf]

Additional file 1:

Receiver operating characteristic curve for maximum standardized uptake values on differentiating between mycobacterial lung disease and lung cancer.

Area under the curve: 0.67.

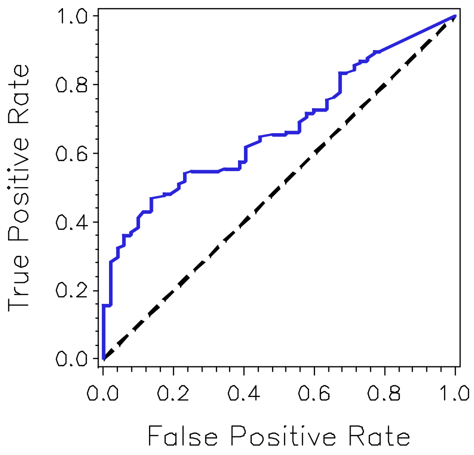

Definitions

		Lung cancer	Mycobacterial lung disease
SUV max cutoff	+	True positive (TP)	False positive (FP)
	-	False negative (FN)	True negative (TN)

Sensitivity= $\frac{TP}{TP+FN}$

Specificity= $\frac{TN}{TN+FP}$

Positive predictive value= $\frac{TP}{TP+FP}$

Negative predictive value= $\frac{TN}{TN+FN}$

Thres hold	True Negative Rate (Specificity)	False Positive Rate (1-Specificity)	True Positive Rate (Sensitivity)	Positive Predictive Value	Negative Predictive Value
1	0.230769	0.769231	0.892086	0.756098	0.444444
2	0.326923	0.673077	0.81295	0.763514	0.395349
3	0.365385	0.634615	0.741007	0.757353	0.345455
4	0.423077	0.576923	0.719424	0.769231	0.360656
5	0.480769	0.519231	0.661871	0.773109	0.347222
6	0.596154	0.403846	0.589928	0.796117	0.352273

Threshold	True Negative Rate (Specificity)	False Positive Rate (1-Specificity)	True Positive Rate (Sensitivity)	Positive Predictive Value	Negative Predictive Value
7	0.653846	0.346154	0.553957	0.810526	0.354167
8	0.788462	0.211538	0.510791	0.865854	0.376147
9	0.865385	0.134615	0.453237	0.9	0.371901
10	0.903846	0.096154	0.381295	0.913793	0.353383
11	0.942308	0.057692	0.338129	0.94	0.347518
12	0.961538	0.038462	0.316547	0.956522	0.344828
13	0.980769	0.019231	0.280576	0.975	0.337748
14	0.980769	0.019231	0.23741	0.970588	0.324841
15	0.980769	0.019231	0.208633	0.966667	0.31677
16	1	0	0.158273	1	0.307692
17	1	0	0.129496	1	0.300578
18	1	0	0.122302	1	0.298851
19	1	0	0.086331	1	0.290503
20	1	0	0.071942	1	0.287293
21	1	0	0.057554	1	0.284153
22	1	0	0.035971	1	0.27957
23	1	0	0.021583	1	0.276596
25	1	0	0.014388	1	0.275132
35	1	0	0.007194	1	0.273684
37	1	0	0		0.272251
